# Bayesian hierarchical spatial regression of maternal depressive symptoms in South Western Sydney, Australia

**DOI:** 10.1186/2193-1801-3-55

**Published:** 2014-01-27

**Authors:** John G Eastwood, Bin B Jalaludin, Lynn A Kemp, Hai N Phung

**Affiliations:** Community Paediatrics, South Western Sydney Local Health District, Locked Mail Bag 7008, Liverpool, BC 1871 New South Wales Australia; School of Public Health and Community Medicine, The University of New South Wales, Sydney, NSW 2052 Australia; School of Women’s and Children’s Health, The University of New South Wales, Sydney, NSW 2052 Australia; School of Public Health, University of Sydney, Sydney, NSW 2006 Australia; School of Public Health, Griffith University, Gold Coast, Queensland, 4222 Australia

**Keywords:** Postnatal depression, Spatial epidemiology, Social capital, Social cohesion, Bayesian hierarchical regression, Exploratory factor analysis

## Abstract

**Background:**

There is increasing interest in the role played by maternal depression in mediating the effects of adversity during pregnancy and poor infant outcomes. There is also increasing evidence from multilevel regression studies for an association of area-level economic deprivation and poor individual mental health. The purpose of the study reported here is to explore the spatial distribution of postnatal depressive symptoms in South Western Sydney, Australia, and to identify covariate associations that could inform subsequent multilevel studies.

**Methods:**

Mothers (n = 15,389) delivering in 2002 and 2003 were assessed at 2–3 weeks after delivery for risk factors for depressive symptoms. The individual-level binary outcome variables were Edinburgh Depression Scale (EDS) >9 and >12. The association between social, demographic and ecological factors and aggregated outcome variables were investigated using exploratory factor analysis and multivariate hierarchical Bayesian spatial regression. Relative risks from the final EDS >12 regression model were mapped to visualise the contribution from explanatory covariates and residual components.

**Results:**

The exploratory factor analysis identified six factors: neighbourhood adversity, social cohesion, health behaviours, housing quality, social services, and support networks. Variables associated with neighbourhood adversity, social cohesion, social networks, and ethnic diversity were consistently associated with aggregated depressive symptoms. Measures of social disadvantage, lack of social cohesion and lack of social capital were associated with increased depressive symptoms. The association with social disadvantage was not significant when controlling for ethnic diversity and social capital.

**Conclusions:**

The findings support the theoretical proposition that neighbourhood adversity causes maternal psychological distress and depression within the context of social buffers including social networks, social cohesion, and social services. The finding have implications for the distribution of health services including early nurse home visiting which has recently been confirmed to be effective in preventing postnatal depression.

**Electronic supplementary material:**

The online version of this article (doi:10.1186/2193-1801-3-55) contains supplementary material, which is available to authorized users.

## Background

There is increasing interest in the role played by maternal depression in mediating the effects of adversity during pregnancy and poor infant outcomes. There is also increasing evidence from multilevel regression studies for an association of area-level economic deprivation and poor individual mental health (Fone et al. 2007; Skapinakis et al. 2005; Weich et al. 2003). Perinatal depression has consistently been found, at the individual level, to be higher among women with low socio-economic status (Beck 2001; O’Hara and Swain 1996). This raises the possibility that the impact of economic and social deprivation on mothers and their infants may be mediated through perinatal depression. With the exception of an area-level study of poverty and postpartum psychosis (Nager et al. 2006) we found no previous published ecological or multilevel studies of perinatal depression.

We have previously reported on individual level psychosocial predictors of perinatal depression in South Western Sydney (SWS) and proposed that the findings were consistent with group-level socioeconomic deprivation, neighbourhood environment, social capital and ethnic diversity having causal effects on postnatal depressive symptomatology and other perinatal outcomes (Eastwood et al. 2011). That proposition is consistent with a recent qualitative study of pathways from neighbourhoods to mental well-being which found that neighbourhood affordability, negative community factors including crime and vandalism, and social makeup including unemployment and poverty, were associated with poor mental wellbeing (O’Campo et al. 2009).

The study reported here uses Bayesian hierarchical modelling techniques to explore spatial relationships between aggregated postnatal depressive symptoms and potential ecological covariates identified in a preceding qualitative study (Eastwood et al. 2014a, Eastwood et al. 2014b). Unlike conventional statistical inference which derives the average estimates of parameters, hierarchical Bayesian modelling produces parameter estimates for each individual analysis unit by borrowing information from all other analysis units (Zhu et al. 2006). Likelihood ordinary least squares (OLS) linear regression treats each suburb in isolation so that there is no recognition in the model of the spatial relationships that may exist between suburbs and therefore no modelling of spatial variability (Law and Haining 2004).

To address this problem Law and Haining (Law and Haining 2004) proposed fitting random effects models where the random variation is spatially structured. A key feature of Bayesian spatial regression models is that they incorporate such random spatial effects into the modelling of outcome and covariate effects at the ecological level. These random spatial effects may reflect unmeasured confounders and thus the model makes it possible to ascertain whether the residual effects suggest spatial patterns or clusters (MacNab 2004).

The study reported here is part of a larger multilevel research programme that utilises critical realist mixed qualitative and quantitative methodology to build a theoretical model of the mechanisms by which multilevel factors might influence the developmental origins of health and disease (Eastwood et al. 2014c). The aim of this study is to explore the spatial distribution of perinatal depressive symptoms in South Western Sydney and to identify any associations that can inform subsequent multilevel studies, theory building and local public health interventions.

## Methods

### Study design

The analysis reported here is part of an exploratory ecological study of aggregated rates of postnatal depressive symptoms in South Western Sydney Area Health Service from 2002–2003. The aggregated rates used in this study were derived from an individual-level cross-sectional study of mothers of infants born in South Western Sydney Area Health Service (SWSAHS) from 2002 to 2003 (Eastwood et al. 2012a). That study (n = 15,389) was a sub-sample from a larger dataset collected from 1998 to 2006. A 2004 to 2006 subsample was retained for subsequent confirmatory studies.

The main study included: individual level logistic regression (Eastwood et al. 2012a) and non-linear principal component analysis (Eastwood et al. 2012b); group-level visualisation of maps of co-variants, cluster analysis (Eastwood et al. 2013a), exploratory factor analysis (Eastwood et al. 2013bEastwood et al. 2014a), ecological likelihood and Bayesian spatial linear regression (Eastwood et al. 2013b); and Bayesian spatial multi-level analysis. The decomposed results of the Bayesian spatial linear regression are reported here using the findings for the previously reported EFA to assist interpretation.

### Study setting

The setting is all suburbs in four local government areas (LGAs) south-western Sydney, New South Wales (NSW), Australia. The individual-level data available for study was coded by suburb of residence. The suburb of residence was chosen as the closest group-level administrative unit to naturally occurring local neighbourhood environments. There were 101 suburbs available to study using the 2001 Census maps.

### Participants

As previously reported the main study utilised the Ingleburn Baby Information System (IBIS) database. That database was initiated in 1995 and is based on the routine survey by Child and Family Health Nurses of all mothers who attend the first well baby clinic (home visit or clinic based) after discharge from the post-natal ward. Population-based collection started in Campbelltown and Wollondilly in 1998, followed by Bankstown in 2000, Fairfield and Wingecarribee in 2001 and Liverpool in 2002. The calendar years of 2002 and 2003 were used for this study as all geographical areas, and 92% of births (n = 21,991) were surveyed. Of those surveyed 70 percent consented to completing an EDS and were included in this analysis. The mothers who did not complete an EDS were more likely to report: difficult financial situation, public housing accommodation, low maternal education, not breast feeding and short suburb duration. We have reported previously on the systematic nature of the missing data in the 2002 to 2003 subsample used for our exploratory studies (Eastwood et al. 2012a).

### Outcome variable

The outcome variable used for this study is the Edinburgh Depression Scale (EDS) (Cox et al. 1987), which has been widely used to study individual maternal perinatal depressive symptoms. This study reports on group level aggregation of two individual-level binary outcome variables, namely for EDS > 9 and EDS > 12 (Eastwood et al. 2012a). The use of EDS > 9 and EDS > 12 are supported by previous studies as screening cut-off points in English-speaking populations (Cox et al. 1987; Buist et al. 2002). Buist et al. ( 2002) noted that using EDS > 12 was a sound option for reducing false positive diagnosis of depressive illness. The original authors recommended using the EDS > 9 as a community screening cut-off point as many women experiencing considerable dysfunction, but not meeting diagnostic criteria for formal illness, might otherwise be missed. Those women also merit recognition of their distress and the provision of psychological or social assistance (Buist et al. 2002; Brown et al. 2001).

### Covariates

As previously reported (Eastwood et al. 2013b, Eastwood et al. 2014a), the selection of group level variables for analysis was principally influenced by concepts emerging from interviews with experienced maternal and child health practitioners, group interviews with mothers of infants and latent variables identified in individual level nonlinear principal component analysis (Eastwood et al. 2012b). The domains assessed as measurable at the suburb level were: social networks, capital and cohesion; “depressed community”, health behaviours, access to services and ethnic segregation or integration.

2001 Census data were used for the majority of the group level candidate variables. NSW crime statistics and aggregated individual level study data were also used. Areal data that was not available at the suburb level could not be used for the analysis reported here. Forty six variables were selected as candidate variables and have been previously described (Eastwood et al. 2013bEastwood et al. 2014a). A table summarising the 46 variables is available as an on-line supplement to this paper (Additional file 1: Table S1).

Selected variables are described here to aid interpretation. The Index of Relative Socioeconomic Disadvantage (IRSD) is a composite index that contains indicators of disadvantage such as low income, high unemployment and low levels of education. The IRSD reflects lack of disadvantage rather than advantage and a high score implies than an area has few families with low income, few people with little or no training and few people working in unskilled occupations (ABS 2001).

For concepts such as social cohesion and social capital there were no candidate variables in the 2001 Census data. Voluntary work was used from the 2006 Census. Social capital questions from the NSW Health survey were only available at local government level. We therefore used the aggregated IBIS variables “social support” and “no regret leaving [the] suburb”, while acknowledging the possibility of same-source bias as identified by Radenbush and others (Duncan and Raudenbush 1999; Radenbush and Sampson 1999) cited by (O’Campo 2003).

As previously observed the multicultural nature of the population of the study area and themes emerging from the qualitative studies necessitated the selection of measures of ethnic diversity (Eastwood et al. 2013a Eastwood et al. 2013b Eastwood et al. 2013c Eastwood et al. 2014a Eastwood et al. 2014b). Three measures of ethnic diversity were selected based on the advice given to the US Bureau of the Census by Galster 2004. They were the: Maly Neighbourhood Diversity Index (Maly 2000), Entropy Index (Modarres 2004) and the Simpson’s Index (Simpson 1949). The Maly (neighbourhood diversity) index ranches from 0 – 1 where a suburb is: 0 with a completely homogeneous ethnic group and, 1 with a completely heterogeneous distribution which matches the metropolitan distribution. The Simpson and Entropy indexes are similar with 0 being completely homogenous and 1 being completely heterogeneous distribution of ethnic groups.

The qualitative interviews also drew attention to the possible importance of extremes of wealth and poverty within neighbourhoods. As previously reported (Eastwood et al. 2013b Eastwood et al. 2014a), we used here the Index of Extremes (ICE) which is computed as the number of affluent families in Census tract minus the number of poor families, divided by the total number of families in the Census tract (Massey 2001; Casciano and Massey 2008). The index varies from a theoretical minimum of -1.0 (all families are poor) to a theoretical maximum of +1.0 (all families are affluent) and passes through 0 (affluent and poor are equally balanced).

### Statistical analysis

Forty six candidate variables (Additional file 1: Table S1) were analysed using correlation matrices, univariate likelihood OLS linear regression, and factor analysis related diagnostic tests of multi-collinearity. Examination of the initial correlation matrices identified variables that were highly correlated and some were mutually exclusive. For example “different address five years previously” and “same address five years previously”. Of the initial 46 variables 31 were selected for EFA following the initial analysis. The EFA then commenced with a serious of further diagnostics as described below. At the completion of the diagnostics the number of variables entered into the EFA had been reduced to 26.

### Factor analysis

As observed above the aim of the main study was theory building. For theoretical EFA where there is expected to be correlation between factors the most appropriate method of extraction is “common factor analysis” as opposed to principal component analysis. As previously reported we used principal-axis factoring with oblimm oblique rotation. We also compared the solution with outputs from Principal Component Analysis (PCA) and EFA with orthogonal rotation. The extracted factors were in all cases the same.

The initial correlation matrix was examined for high correlations among variables. Bartlett’s test, the Kaiser-Meyer-Olkin measure, and examination of the anti-image matrix were also used to evaluate whether the correlation matrix was suitable for factor analysis. Communalities that loaded less than 0.50 were also analysed.

We generated a scree plot to determine the adequate number of components to retain in the analysis. After comparing extractions of 5, 6, 7 and 8 solutions, a 6 factor solution was selected as the best in terms of interpretability.

The exploratory factor analysis enabled identification of multi-colinearity among the candidate covariates, assisted in the selection of variables for analysis in the spatial regression models and identified underlying latent variables for subsequent theory building.

### Bayesian modelling EDS

The Bayesian modelling strategy was to first fit a log-normal model for relative risk using observed and expected counts for both of the outcome variables EDS > 9 and EDS > 12. The expected count for each suburb was computed using the observed EDS > 9 or EDS > 12 rates for the South Western Sydney region multiplied by the number of mothers surveyed in that suburb. The number of women surveyed in each suburb may not have represented the true distribution of women who might have been surveyed. We therefore also undertook standardisation using “women of child bearing age” (WCBA). The rates standardised by numbers surveyed was highly correlated with the standardisation by WCBA and we therefore used the numbers surveyed.

This basic log-normal model allowed for subsequent covariate adjustment, for spatial correlation of risk in nearby areas and for the addition of unstructured variance terms. This ‘Besag, York and Mollie’ (BYM) model for relative risks, (Besag et al. 1999) takes into account the effects that vary in a structured manner in space (clustering or correlated heterogeneity) and a component that models the effects that vary in an unstructured way between areas (uncorrelated heterogeneity) (Lawson et al. 2003).

The conditional autoregressive (CAR) component used data from an adjacency matrix for the study area which was generated using the Adjacency Tool in GeoBUGS 1.1 (Spiegelhalter et al. 2003). We used the map decomposition strategy developed by Law and Haining (Law and Haining 2004) to visualise the results of the best fitting models. The map decomposition method allows separate mapping of the contribution made by the explanatory covariate variables, spatial clustering and heterogeneity (see Additional file 2: Table S2 for the WinBUGS code).

Each covariate was fitted separately and model fit was assessed using changes in deviance information criterion (DIC). We also report the pD which is the number of effective parameters in the model. Smaller values of DIC indicate better fitting models. To compare DIC values, we carried out several runs using different initial values and random-number seeds (Spiegelhalter et al. 2002). Based on the results, we chose to distinguish between models where the DIC varied by more than one.

Significance was assessed as non-zero regression coefficients at 95% credible interval. The covariate, that when added, resulted in the largest decrease in DIC was then analysed together with each of the remaining covariates. The process was repeated by adding further covariates until the DIC could be reduced no further. The final model(s) are reported.

The specification of priors for the parameters is important in Bayesian inference. There have been no previously reported Bayesian studies of postnatal depressive symptoms; and therefore for the intercept we used a “vague” prior with a normal distribution, a mean of zero and large variance *N*(0, 10 000). For the precision of the random effects we used the commonly used hyper-prior of Gamma (0.5, 0.0005) as recommended by Kelsall and Wakefield (Kelsall and Wakefield 2002). For the variance we used an inverse Gamma prior. Sensitivity analysis was undertaken.

All models assessed were run using two chains simultaneously. Convergence had occurred by 3,000 iterations in all cases. Each chain was then run for a further 3,000 iterations, with acceptable Monte Carlo (MC) errors. These samples were used to generate the posterior distributions from which the estimates of the parameters were obtained.

The exploratory factor analysis was undertaken using SPSS 17.00. All Bayesian analysis was undertaken in WinBUGS 1.4 (Spiegelhalter et al. 2003). The posterior relative risks were mapped using ArcGIS 3.2 (Environmental Systems Research Institute 2008).

### Ethics approval

The study obtained ethics approval from the Human Research Ethics Committee, South Western Sydney Area Health Service and from the University of NSW Human Research Ethics Committee.

## Results

### Exploratory factor analysis (EFA)

We have previously reported the results of the factor analysis (Eastwood et al. 2013b Eastwood et al. 2014a). The pattern matrix factor loadings obtained are shown in Table 1 to assist the later theoretical analysis of the Bayesian spatial regression. The factor loadings arranged in decreasing order within factors and with loadings less than 0.30 suppressed to aid interpretation.

**Table 1:** **Factor analysis oblique rotation output – pattern matrix**

	Factors					
	1	2	3	4	5	6
One Parent Families%	.912					
Rented Dwellings%	.818			-.360		
Public Housing%	.777					
Unplanned Pregnancy%	.709					
Occupational Class 3%	.584	-.437				
Poor Families%	.567					
Violent Crime rate	.546					
Volunteerism%		.927				
Entropy Index		-.784		-.307		
No volunteerism%		-.651		.305		
Low Schooling%		-.567				-.406
IRSD Decile	-.468	.528				
Breast feeding%			.907			
Smoking%			.899			
No Regret Leaving%			.795			
Apartments%				-.825		
Single Houses%	-.427			.728		
Vacancy Rate				-.637		
Nurse Visit Rate		.405			.767	
Home Visit Rate					.535	
Poor Health%	.304				.363	
No Social Support%						-.679
No Practical Support%						-.589
Density		-.452				-.493
Different address last 5 years					.349	.395
Maly Index						.308

Extraction method: principal axis factoring.

Rotation method: oblimin with Kaiser Normalization.

Based on the loadings in Table 1 we have here called Factor 1 – *disadvantaged community*; Factor 2 – *Social Cohesion;* Factor 3 - *Health Behaviours*; Factor 4 - *Housing Quality*; Factor 5 – *Access to Services*; and Factor 6 – *Social Capital.*

### Spatial linear regression (Bayesian)

We first fitted a log-normal model followed by models with uncorrelated and correlated components. Models with only spatial random effects were the best fit closely followed by the full ‘Besag, York and Mollie’ (BYM) model. We then fitted every candidate variable using the spatial random effects model. Table 2 shows the effective number of parameters in the model (*p*D) and the Deviance Information Criteria (DIC) results for models with: intercept only, correlated and uncorrelated residuals, full BYM, and a representative set of covariates.

**Table 2:** **Comparison of DIC for selected covariates in univariate spatial models**

	EDS > 9		EDS > 12	
Variable	pD	DIC	pD	DIC
Intercept Only	1.001	601.734	0.981	502.008
+ uncorrelated residual	28.285	580.712	21.814	487.733
+ spatial residual (CAR)	23.623	565.503	21.957	473.102
+ spatial + uncorrelated residual	26.185	567.673	23.887	475.944
**CAR with covariates**				
One Parent Family%	23.082	565.160	18.677	472.724
Rented Dwelling%	23.162	564.836	17.747	472.030
Unplanned Pregnancy%	18.828	561.784	13.814	470.470
Poor Families%	21.602	564.252	15.218	471.154
Volunteerism%	15.460	561.749	13.158	468.012
Entropy Index	17.593	564.483	14.551	469.984
Low Schooling%	18.680	562.840	15.848	471.488
IRSD Decile	13.635	564.481	7.529	472.089
Breastfeeding%	22.781	565.391	19.899	473.967
No Regret Leaving%	18.237	562.061	13.800	465.737
Apartments%	24.575	566.794	17.558	471.806
Nurse Visit Rate	25.430	567.276	22.230	475.631
No Social Support%	15.907	560.012	11.433	466.878
Maly Index	23.994	564.131	19.074	470.150
Density	18.318	561.917	15.545	470.901

Legend: EDS (Edinburgh Depression Scale), IRSD (Index of Relative Social Deprivation), CAR (Conditional Autoregression), pD (Effective number of parameters in the model), DIC (Deviance Information Criteria).

### Best fitting spatial models

The multivariate EDS > 9 model with the lowest DIC and no non-significant coefficients included the covariates:% No Social Support, Entropy Index,% Apartments and% Smoking (Table 3). Models that included:% No Regret Leaving and% One Parent Families also performed well.

**Table 3:** **Best fitting spatial CAR regression models**

Models	Pd	DIC
EDS > 9 Best Fitting CAR Models		
No Covariate CAR Model	23.404	565.468
No Support	15.907	560.012
No Support + Entropy	13.012	558.326
No Support + Entropy +%Apartment	12.373	554.417
No Support + Entropy +%Apartment + nurse visit rate	10.449	555.714
No Support + Entropy +%Apartment +%No regret leaving	10.146	554.072
No Support + Entropy +%Apartment +%One Parent Families	9.296	553.803
No Support + Entropy +%Apartment +%Smoking	10.543	552.535
No Support + Entropy +%Apartment +%Smoking (BYM)	12.340	553.200
EDS > 12 Best Fitting CAR Models		
No Covariate	21.481	473.277
No Support	11.433	466.878
No Support + Entropy	10.352	462.743
No Support + Entropy + Smoking	7.997	460.240
No Support + Entropy + No Regret Leaving Suburb	9.208	459.622

The multivariate EDS > 12 models with the lowest DIC and no non-significant coefficients included:% No Social Support, Entropy Index, and either% No Regret Leaving or% Smoking (Table 3). There were no other models with three covariates where the coefficients were significant.

We have elected to report here in more detail the best fitting of the aggregated EDS > 12 models namely that including the covariates:% No Support, Entropy Index and “%No Regret Leaving”. Table 4 shows the summary posterior parameters for the final EDS > 12 BYM model. Table 5 shows the posterior Odds Ratio for the final EDS > 12 BYM model. The odds of EDS > 12 was increased in suburbs with increased percentage of “no support” and percent “No Regret Leaving”. The odds was also increased in suburbs with a high Entropy Index.

**Table 4:** **Summary statistics for parameters in BYM model EDS >12**

Node	Mean	sd	MC error	2.50%	Median	97.50%
Intercept	-0.098	0.037	0.000	-0.171	-0.098	-0.026
No Support	0.074	0.035	0.001	0.004	0.074	0.142
Entropy Index	0.112	0.047	0.001	0.021	0.112	0.205
No regret leaving	0.143	0.057	0.001	0.030	0.144	0.253

Legend: sd (standard deviation), MC (Monte Carlo).

**Table 5:** **Posterior expected odds ratio for BYM model EDS > 12**

Node	Mean	sd	MC error	2.50%	Median	97.50%
OR.%No Support	1.08	0.04	0.00	1.00	1.08	1.15
OR. Entropy Index	1.13	0.05	0.00	1.03	1.13	1.23
OR.%No Regret Leaving	1.15	0.07	0.00	1.03	1.15	1.28

Legend: sd (standard deviation), MC (Monte Carlo).

Map decomposition of the full BYM model enables visualisation of the full range of posterior estimated relative risks and residuals. This approach allows high and low areas of relative risk to be identified as well as an assessment of the contribution made by the covariates, unstructured and spatially structured random effects.

**Figure 1:**
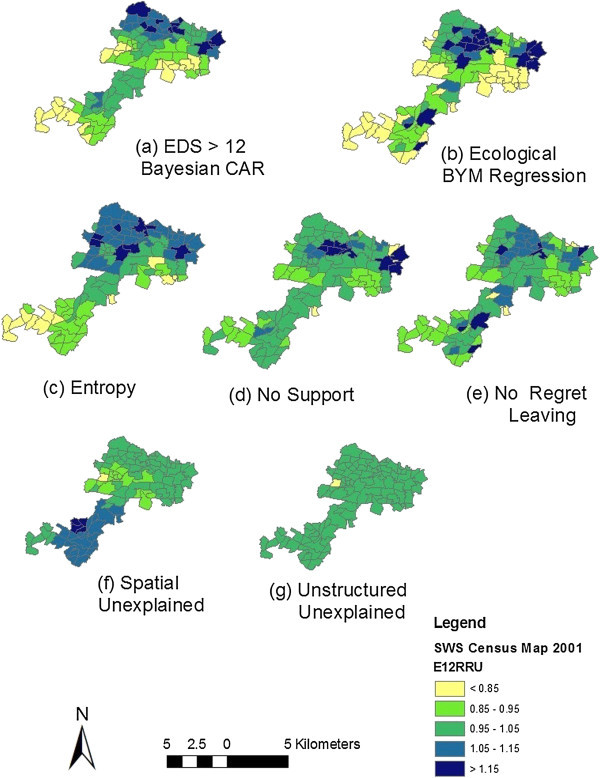
**Posterior expected relative risks (BYM model) for EPDS > 12. (a)** EDS > 12 Bayesian CAR; **(b)** Ecological BYM Regression; **(c)** Entropy fixed effect; **(d)** No support fixed effect; **(e)** No regret leaving fixed effect; **(f)** spatial unexplained component; **(g)** unstructured unexplained component.

Figure 1 shows the map of Relative Risk for EDS > 12 in the final BYM model. Clustering of EDS > 12 can be seen in the northern suburbs in the “basic” CAR model. The relative risks (RRs) for areas in the north are strongly driven by the covariates “% No Social Support”, and Entropy. The covariate% No Regret Leaving is making a contribution to the RR in several other areas of the map.

The maps of the residuals are strongly dominated by the unexplained spatial residual which is strongest in the southern suburbs.

## Discussion

The analysis presented here has identified suburbs in South Western Sydney where rates of maternal depressive symptoms are higher than expected for the region as a whole. We know from previous spatial studies of South Western Sydney (Phung et al. 2003) that those same communities have higher rates of social disadvantage and non-English speaking populations. Our qualitative studies also indicated that maternal depressive symptoms would be higher in communities with weak social networks, capital or cohesion; “depressed community”, poor access to services and ethnic segregation.

Figure 1a shows the RRs for EDS > 12 in a model with no covariates. Clustering of EDS > 12 can be seen in the north eastern and north western suburbs. The pattern in the final BYM model is different with high RRs in more central suburbs of the northern region and in a few southern suburbs. The map decomposition indicates which of the covariates contribute to the ecological risk of depressive illness and in which suburbs. The RRs for areas in the north are strongly driven by the covariates “% No Support” and Entropy, while the covariate% No Regret Leaving is making a contribution to the RRs in several other areas of the map.

The covariate% No Support is an aggregate variable of no support network at the individual level. We consider that this variable, and Factor 6 on which it loads, represents the latent variable *social capital* at the suburb level. The Entropy Index is a measure of ethnic diversity which loaded on Factor 2. In this study it is associated with a latent variable (F2) which may represent *social cohesion*. In the factor analysis, social cohesion was only slightly correlated with social capital (r = 0.26). The spatial distribution (not reported here) of the factors is also different. Together with the spatial regression analysis reported here, these findings support the proposition that social cohesion and social capital are independent latent variables that are negatively associated with aggregated rates of depressive illness.

The variable “no regret at leaving the suburb” has been used in previous studies as an indicator of social cohesion (NSW Department of Health 2006). In our study, this variable is correlated with community norms of smoking and not breastfeeding and not related to either *social cohesion* or *social capital*. In the EFA correlation matrix, *health behaviours* (F3) was moderately correlated with *disadvantaged community (r = 0.4)* suggesting that suburbs with poor “health behaviours” were also likely to be disadvantaged.

Interestingly, there were no variables from Factor 1 representing the possible latent variable of “disadvantaged community” in the final EDS > 12 model. One of the final EDS > 9 regression models included% One Parent Families. The univariate spatial regression identified variables loading on Factor 1 as significantly associated with aggregated depressive symptoms, including: Index of Relative Social Deprivation (IRSD),% Occupation Class 3,% unemployed,% One Parent Families,% poor families and Index of Concentrated Extremes (ICE). When controlling for entropy and% no support in the EDS > 12 model, these variables were no longer significant. Aneshensel ( 2009) notes that distal factors, such as income, are often not statistically significant when more proximal factors, such as social support, are taken into consideration. The findings here would be consistent with the latent variable *disadvantaged community,* and its associated observed variables, being distal *to social cohesion* and *social capital* on a causal pathway as proposed by Carpiano ( 2006).

The maps of the residuals are strongly dominated by the unexplained spatial component which is strongest in the southern suburbs. The implication of this is that a covariate that would remove this spatial residual has not been included in the model. It is also possible that such a covariate was not identified among the candidate variables included in this study. The study reported here was of all mothers giving birth in 2002 to 2003. The spatial residuals may also represent a sub-group of mothers who are at higher risk when living in the southern suburbs where social support networks are strong.

The findings of the spatial regression could be interpreted as suggesting that mothers were more likely to have depressive symptoms if they live in communities with low social support networks, ethnic diversity (heterogeneity) and populations who would have “no regret leaving”. Based on the factor analysis loading of these three variables the findings suggest that women are more likely to be depressed if they live in communities with low social capital, low social cohesion and community-level unhealthy behaviours (which are associated with disadvantaged communities).

### Methodological matters

As we have previously observed (Eastwood et al. 2012a), that the size (15,389) of this cross-sectional study of the EDS administered to postnatal women is unique. There have been few previous reports of postnatal depression studies on population samples of this order. We have also previously reported on the limitations of the cross-sectional design for causal inference and the impact on generalizability of sample selection bias from refusal and non-response in the study population. Importantly not all households with births in the study period were surveyed. That would have included mothers who had lost infants. The households not questioned with the IBIS questionnaire included mothers who moved to “out of area” locations or mothers who declined the nurse first-visit offered. Also missing may have been some infants in intensive care units.

Observational (information) bias may have been present in the survey data. This could have arisen from recall bias, or interviewer/responder bias. A particular problematic feature of self-reporting surveys is the mental state of the subject. Depressed women are more likely to have a negative view of their circumstances. This must be taken into account when considering the association found in this study of high EDS with subjective variables such as “rating of own health”, “reluctance to leave the suburb”, and “difficult financial situation”.

The EDS is administered in English or via an interpreter where the mother is non-English speaking (NESP). We were not able to report in this study on the percentage of NESP mothers in the full sample but the percentage of NESP mothers in the Local Government Areas of Fairfield, Campbelltown, Camden and Wollondilly (linked data) was 11.8 percent at the time of the 2001 Census. This may be an important source of information bias in this study. Specific non-English EDS are not currently used postpartum in SWS but could be considered for future use.

The limitations of ecological studies in making inferences regarding individual-level associations based on group-level data are well established (Greenland 1992). The findings reported in this study could reflect a compositional effect reflecting the aggregated characteristics of women (and possibly their partners) with depressive symptoms. Multilevel studies enable the simultaneous examination of the effects of group level and individual level variables on individual level outcomes (Duncan et al. 1995). Causal inference would be assisted by such an approach.

The strength of the current study is the use of Bayesian hierarchical approaches to spatial autocorrelation thus addressing the limitations of linear methods which treat each suburb as being independent of surrounding suburbs. The map decomposition strategy developed by Law and Haining ( 2004) proved useful and in particular raised questions regarding the spatial distribution of unexplained components. Bayesian methods are increasingly being used in multilevel studies but their use in spatial multilevel studies has been limited. The findings of our Bayesian multilevel spatial studies will be reported separately.

## Conclusion

This study found significant variation between suburbs in relation to maternal depressive symptoms. The variation was associated with differences in neighbourhood socioeconomic status, social cohesion, social capital, and ethnic diversity. These findings support the proposition that social stratification is associated with mental health disparities. The finding have implications for the distribution of health services including early nurse home visiting which has recently been confirmed to be effective in preventing postnatal depression (Morrell et al. 2009).

## Electronic supplementary material

Additional file 1: Table S1: Candidate variables. (DOC 60 KB)

Additional file 2: Table S2: WinBUGS Code for full GYM spatial model EDS > 9 with map decomposition. (DOCX 19 KB)

## Abbreviations

EDS: Edinburgh Depression Scale
OLS: Ordinary least squares
BYM: Besag, York and Mollie’
pD: Effective number of parameters in the model
DIC: Deviance Information Criteria
RR: Relative risk
EFA: Exploratory factor analysis
IRSD: Index of Relative Social Deprivation
ICE: Index of Concentrated Extremes
NESP: non-English Speaking
SWS: South Western Sydney
SWSAHS: South Western Sydney Area Health Service
NSW: New South Wales
IBIS: Ingleburn Baby Information System
PCA: Principal Component Analysis
WBCA: Women of Child-bearing Age
CAR: Conditional Auto-regression
MC: Monte Carlo.
