# Supplementary material for: Bayesian hierarchical spatial regression of maternal depressive symptoms in South Western Sydney, Australia
Source: Springerplus. 2014 Jan 27;3:55. doi: 10.1186/2193-1801-3-55 (PMC3921342; doi:10.1186/2193-1801-3-55)
Supplement: Supplementary file 1 — Additional file 1: Table S1: Candidate variables. (DOC 60 KB) [file 40064_2013_808_MOESM1_ESM.doc]

Additional file 1: Table S1. Candidate variables

| **Variable Name** | **Description** | **Concept Selected for** | **Type/ Source** |
| --- | --- | --- | --- |
| Density | Total population per square kilometre (2001 Census) | Depressed Community | Integral |
| Entropy Index | Entropy Index (2001 census) | Ethnic | Derived |
| Entropy Log Index | Normalised Log of Entropy Index | Ethnic | Derived |
| Simpson Index | Simpson Index | Ethnic | Derived |
| Maly Index | Maly Index | Ethnic | Derived |
| Volunteerism | Voluntary work by total population > 15 years (2006 Census) | Social Capital | Derived |
| No Volunteerism | No Voluntary work by total population > 15 years (2006 Census) | Social Capital | Derived |
| Universal Nurse Visit Rate | Average number of first nurse visit, 2002-2003, by infants resident, 2001 Census | Access | Derived |
| Universal Home Visit Percent | Average number of first nurse visit, 2002-2003, by infants resident, 2001 Census | Access | Derived |
| Nurse Visit Rate | Average number of all nurse visits, 2002-2003, by infants resident, 2001 Census | Access | Derived |
| Poor Families | 50 percent of NSW median family weekly income, 2001 Census. Number of families with weekly income $0-$499 per week by total families | Depressed Community | Derived |
| Rich Families | Percentage of with income greater than the NSW median family income, 2001 Census. Number of families with weekly income greater than $800-$999 per week | Depressed Community | Derived |
| ICE | Number of rich families minus number of poor families divided by the total families per suburb (2001 census) | Depressed Community | Derived |
| Smoking | Percent of mothers smoking at first home visit (IBIS 2002-2003) | Suburb norm of health behaviour | Derived |
| Unplanned Pregnancy | Percent of mothers who planned the pregnancy (IBIS 2002-2003 | Suburb norm of health behaviours | Derived |
| Public Housing | Percent of families in public housing (IBIS 2002-2003) |  | Derived |
| Breastfeeding | Percent of mothers breast feeding (IBIS 2002-2003) | Suburb norm of health behaviours | Derived |
| No Social Support | Percent of mothers with no social support network (IBIS 2002-2003) | Social Capital | Derived |
| Social Support | Percent of mothers with social support network (IBIS 2002-2003) | Social Capital | Derived |
| No Practical Support | Percent of mothers with no practical support network (IBIS 2002-2003) | Social Capital | Derived |
| Practical Support | Percent of mothers with practical support network (IBIS 2002-2003) | Social Capital | Derived |
| No Emotional Support | Percent of mothers with no emotional support network (IBIS 2002-2003) | Social Capital | Derived |
| Emotional Support | Percent of mothers with emotional network (IBIS 2002-2003) | Social Capital | Derived |
| No Regret Leaving | Percentage of mothers who would not regret leaving the suburb | Social Capital | Derived |
| Regret Leaving | Percent of mothers who would regret leaving the suburb (IBIS 2002-2003) | Social Capital | Derived |
| Poor Health | Percent of mothers reporting poor health (IBIS 2002-2003) | Outcome | Derived |
| Good Health | Percent of mothers reporting good health (IBIS 2002-2003) | Outcome | Derived |
| Owned Dwelling | Percent of owner occupied dwellings (2001 Census) | Depressed Community | Integral |
| Rented Dwellings | Percent of rented dwellings (2001 Census) | Depressed Community | Intregral |
| Unemployment | Percent unemployment (2001 Census) | Depressed Community | Derived |
| Different address last 5 years | Percent of families who lived at a different address 5 years previous(2001 Census) | Social Capital | Derived |
| Same Address last 5 years | Percent of families who lived at the same address 5 years previous (2001 Census) | Social Capital | Derived |
| Different Address last 1 year | Percent of families who lived at a different address 1 years previous (2001 Census) | Social Capital | Derived |
| Vacancy Rate | Percent of vacant dwellings (2001 Census) | Depressed Community | Derived |
| Apartments | Percent of dwellings as apartments (2001 Census) | Depressed Community | Integral |
| Single Houses | Percent of dwellings as single dwelling houses (2001 Census) | Depressed Community | Integral |
| High Apartments | Percent of dwellings as multistorey apartments (2001 Census) | Depressed Community | Integral |
| Occupational Class 3 | Blue collar working occupation class rate (2001 Census) | Depressed Community | Derived |
| Occupational Class 2 | Administration and retail occupation class rate (2001 Census) | Nil | Derived |
| Occupational Class 1 | White collar occupation class rate(2001 Census) | Depressed Community | Derived |
| Occupational Extremes | Index of Extremes of occupation class (2001 Census) | Depressed Community | Derived |
| One Parent Families | Percent of one parent families(2001 Census) | Depressed Community | Derived |
| Low Schooling | Percent of individuals with no schooling or less than year 9 (2001 Census) | Depressed Community | Derived |
| IRSD | Index of Relative Social Disadvantage(2001 Census) | Depressed Community | Derived |
| IRSD Decile | Decile of Index of Relative Social Disadvantage (2001 Census) | Depressed Community | Derived |
| Violent Crime | Violent Crime Rate. Reported violent crime rate at post code level applied to relevant suburbs (NSW Crime Bureau) | Depressed Community | Integral |
