# Supplementary material for: Bayesian hierarchical spatial regression of maternal depressive symptoms in South Western Sydney, Australia
Source: Springerplus. 2014 Jan 27;3:55. doi: 10.1186/2193-1801-3-55 (PMC3921342; doi:10.1186/2193-1801-3-55)
Supplement: Supplementary file 2 — Additional file 2: Table S2: WinBUGS Code for full GYM spatial model EDS > 9 with map decomposition. (DOCX 19 KB) [file 40064_2013_808_MOESM2_ESM.docx]

**Additional file 2: Table S2. WinBUGS Code for full GYM spatial model EDS > 9 with map decomposition**

# Spatial model EDS9 for map decomposition full GYM

model

{

for (i in 1:N) {

EDS9_OBSER[i] ~ dpois(mu[i]);

log(mu[i]) <- log(EDS9_EXPEC[i]) + alpha[1] + NSPT[i]*alpha[2] +ENTROPY[i]*alpha[3]+HAPP[i]*alpha[4]+SMOKE[i]*alpha[5] + s[i] + u[i];

u[i] ~ dnorm(0, tau.u);

#Relative Risk area-specific for maps

RR[i] <- exp(alpha[1] + NSPT[i]*alpha[2] + ENTROPY[i]*alpha[3] + HAPP[i]*alpha[4] + SMOKE[i]*alpha[5] + s[i] + u[i]);

#Relative Risk decomposition

RR_Res[i]<-exp(alpha[1] + s[i] + u[i]);

RR_Spat[i]<-exp(s[i])

RR_Het[i]<-exp(u[i])

RR_NSPT[i]<-exp(alpha[2]*NSPT[i])

RR_ENTROPY[i]<-exp(alpha[3]*ENTROPY[i])

RR_HAPP[i]<-exp(alpha[4]*HAPP[i])

RR_SMOKE[i]<-exp(alpha[5]*SMOKE[i]) }

# CAR prior distribution for random effects:

s[1:N] ~ car.normal(adj[], weights[], num[], tau.s);

for(k in 1:sumNumNeigh) {

weights[k] <- 1; }

# Other priors:

alpha[1] ~ dflat();

for (k in 2:5){

alpha[k] ~ dnorm(0,0.0001)

OR.alpha[k]<-exp(alpha[k]) }

tau.s ~ dgamma(0.5, 0.0005); # prior on precision

sigma.s <- sqrt(1 / tau.s); # standard deviation

tau.u ~ dgamma(0.5, 0.0005); # prior on precision

sigma.u <- sqrt(1 / tau.u); # standard deviation

}

# Initial values

list(alpha=c(0,0,0,0,0),tau.s=50,tau.u=10)

list(alpha=c(0.05,-0.05,0.05,-0.05,0.05),tau.s=50,tau.u=10)
